# MACC1 Is Associated With Epithelial–Mesenchymal Transition and Can Predict Poor Prognosis in Nasopharyngeal Carcinoma

**DOI:** 10.3389/fonc.2021.644120

**Published:** 2021-03-29

**Authors:** Hao Cheng, Linxiang Zhou, Yalan Long, Juanjuan Xiang, Longhua Chen

**Affiliations:** ^1^ Department of Radiation Oncology, Nanfang Hospital of Southern Medical University, Guangzhou, China; ^2^ Department of Nasopharyngeal Carcinoma, The First People’s Hospital of Chenzhou, Southern Medical University, Chenzhou, China; ^3^ Department of Nasopharyngeal Carcinoma, The First People’s Hospital of Chenzhou, University of South China, Chenzhou, China

**Keywords:** metastasis-associated in colon cancer 1 (MACC1), nasopharyngeal carcinoma, epithelial-mesenchymal transition (EMT), vimentin, E-cadherin, prognostic biomarker

## Abstract

**Background:**

Given the reported correlation between the oncogene metastasis-associated in colon cancer 1 (MACC1) and nasopharyngeal carcinoma (NPC), as well as between MACC1 and epithelial–mesenchymal transition (EMT), we speculated that EMT is a likely causative link between MACC1 expression and poor NPC prognosis. Thus, we aim to clarify the relationship between MACC1 and EMT in NPC prognosis.

**Material and Methods:**

We performed immunohistochemical examination of tissue sections from 128 NPC patients that were divided into six groups corresponding to high and low protein expression of MACC1 and two EMT-related proteins, vimentin and E-cadherin, and Kaplan–Meier (KM) survival analyses were performed.

**Results:**

KM survival analysis showed that upregulation of MACC1 and vimentin and downregulation of E-cadherin were significantly associated with reduced survival in NPC. Short hairpin RNA (shRNA) interference and immunoblotting in the NPC cell line HNE-1 led to increased E-cadherin but decreased vimentin levels. MACC1 overexpression was significantly correlated with poor 5-year overall survival, metastasis-free survival, and disease-free survival (P<0.05) but not with poor relapse-free survival (P>0.05). Univariate analyses revealed that MACC1, E-cadherin, and vimentin levels along with T and N tumor classifications and cancer staging are significant prognostic factors of NPC (P<0.05).

**Conclusion:**

Our findings showed the association between MACC1 and EMT in NPC malignancy and support the role of MACC1 as a prognostic biomarker and molecular target for NPC treatment.

## Introduction

Nasopharyngeal carcinoma (NPC) is a malignant tumor that originates from the epithelial cells of the nasopharynx, a region at the bottom of the skull and back of the throat. It is highly prevalent in Southeast Asia, Southeastern China, North Africa, the Middle East and the Arctic (1, 2). Individuals with a history of Epstein-Barr virus (EBV) infection, certain lifestyle and diet habits, and a genetic predisposition due to familial inheritance have been found to exhibit a higher risk of NPC (2, 3). NPC tumors are classified into three types: keratinizing squamous cell carcinoma (type I), non-keratinizing squamous carcinoma (type II), and undifferentiated carcinoma (type III) (2, 4). In general, patients diagnosed at an early stage of NPC have a more promising overall survival rate than those diagnosed at later stages of the cancer, as with other cancers. Patients with stage IV NPC typically have distant metastases, which is a major cause of treatment failure, along with tumor recurrence (5). A lack in reliable prognosis of cancer outcomes spurs continued research to elucidate new molecular markers and clinical and histopathological features for the early and accurate prediction of patients at high risk for disease progression, distant metastases, and cancer recurrence.

Metastasis-associated in colon cancer 1 (MACC1) protein has been implicated in tumor invasion, migration and in epithelial-mesenchymal transition (EMT) (6). MACC1 has been shown to modulate the HGF/MET (7–10) Akt/β-catenin (11) and MAPK/PI3K/Akt (8–10) signaling pathways in various cancers. Upregulation of MACC1 has been found in colorectal cancer (7, 12), gastric cancer (13–15), liver cancer (16, 17), lung cancer (18), non-small cell lung cancer (19), cervical cancer (20, 21), ovarian cancer (22), NPC (11, 23), and malignant gliomas (24). MACC1’s oncogenic role is further evidenced from MACC1 knockdown experiments (11, 15, 25, 26). The consistency of MACC1 mRNA levels in colorectal and liver cancer (26) makes it a strong candidate marker for NPC prognosis.

MACC1 was also shown to function in mediating EMT, a pathophysiological process characterized by the downregulation of cell-to-cell adhesion proteins (11). EMT involves the conversion of nasopharyngeal epithelial cells into mesenchymal cells that are anti-apoptotic and have the transformational ability to migrate, thus promoting cell invasion and metastasis (27, 28). MACC1 upregulation resulted in decreased epithelial markers such as E­cadherin and increased mesenchymal markers such as vimentin in certain cancers (29, 30), thus promoting EMT, tumor invasion and metastasis (25, 31, 32). Given the correlation between MACC1 and EMT, and between EMT and tumor development (33), we hypothesized that MACC1 could be correlated with EMT in NPC malignancy.

We recruited 128 NPC patients for this study to investigate the relationship between MACC1 and the two EMT-related proteins, vimentin and E-cadherin, in NPC and their roles in NPC patient survival outcome, using immunohistochemistry, and survival analyses.

## Material and Methods

### Patient Information and Follow-Up Protocol

A total of 128 patients newly diagnosed with nasopharyngeal carcinoma (NPC) who received radiotherapy in the First People’s Hospital of Chenzhou, China, between March, 2011 and September, 2012 were recruited. The collected data included clinical features such as age, sex, pathological type, T stage, N stage, TNM stage, and details of follow-up examinations.

The inclusion criteria were as follows: pathologically confirmed NPC, a complete data history, exclusion of distant metastasis, and absence of any other severe underlying disease or history of cancer. Pre-treatment assessments included medical history, physical examination, blood, liver function, renal function, and Epstein-Barr virus (EBV) tests, nasopharyngeal sinus and neck magnetic resonance imaging (MRI), chest-computed tomography (CT) scanning, abdominal B ultrasound, and bone scanning. For MRI of the nasopharyngeal sinuses, T and N stages were assessed according to the 2008 Guangzhou staging system (34). The main histopathological type was undifferentiated carcinoma. All cases were followed up in our inpatient and outpatient hospital departments. Follow-up ranged from the end of radiotherapy to June, 2017. All the patients were followed up once every 3 months during the first year of radiotherapy, followed by one visit every 6 months during the second to fifth year, and one visit after 5 years. Follow-up assessments included physical examination, nasopharyngeal sinus and neck MRI, chest CT scanning, abdominal ultrasound, bone scanning, and blood tests.

### Immunohistochemistry

MACC1, E­cadherin, and vimentin levels in NPC specimens were detected with secondary streptavidin-peroxidase (SP) kits (Beijing Boaosen Biotechnology Co. Ltd.). NPC sections were embedded in paraffin and then fixed with 10% formaldehyde at room temperature for 12-24 h. The 4 µm-thick sections were then deparaffinized, rehydrated using serial ethanol gradient, subjected to heat-induced antigen retrieval, and then inactivated with 3% endogenous peroxidase. Subsequently, the sections were first incubated overnight at 4°C with E-cadherin (1:120 dilution; cat# 20874-1-AP, Shanghai Changjia Biological Co. Ltd.), MACC1 or vimentin (1:100; MACC1: cat# 86290; Vimentin: cat# 10366-1-AP; Shanghai Changjia Biological Co. Ltd.) rabbit polyclonal primary antibodies, followed by rabbit secondary antibody (cat# Sc-2004; Maixin Biotechnology Company) for 30 min at room temperature before visualization under a mounted fluorescence microscope (Olympus). Three histological tissue types were tested: undifferentiated carcinoma of nasopharyngeal tissue (UCNT), differentiated non-keratinizing carcinoma (DNKC), and keratinizing squamous cell carcinoma (KSCC). The tumor types were classified using the TNM malignant tumor staging system, wherein T refers to the size of the primary tumor, N refers to whether the neighboring lymph nodes were metastasized, and M refers to distant metastasis. In addition, the severity of the cancer was grouped into four stages (stages I-IV).

Nuclei were counterstained with hematoxylin, dehydrated with ethanol, transparentized and then paraffinized before mounting. Judgment pre-test positive tablets were used as a positive control, and phosphate-buffered saline (PBS) devoid of antibodies as the negative control. The double-blind method, involving two pathologists without prior knowledge of the specific proteins assessed or their role in NPC, was used to assess the stained sections. The staining intensity and the proportion of positively stained tumor cells were assessed and graded as follows: 0, no staining; 1, light yellow weak staining; 2, yellow moderate staining; 3, brown strong staining. Scores for the stained sections were assigned as follows: 0 (<10% positively stained tumor cells), 1 (10-25% positively stained tumor cells), 2 (26-50% positively stained tumor cells), 3 (51-75% positively stained tumor cells), and 4 (>75% positively stained tumor cells). Protein levels were determined by calculating the staining index: total score = staining intensity score x proportion of positively stained tumor cell score. A total score of ≥3 was used to distinguish high from low protein expression: a total score of <3 indicated low/negative protein expression and a total score of ≥3 indicated high/positive protein expression.

### Cell Line and Culture

The NPC cell line HNE-1 (Shanghai Cell Bank of the Chinese Academy of Sciences, China) was cultured in RPMI-1640 medium containing 10% fetal bovine serum (FBS), 100 U/ml penicillin and streptomycin at 37°C in a 5% CO_2_ humidified incubator. HNE-1 cells at 80-90% confluency were harvested for western blot analysis.

### Lentiviral shRNA Transfection

HNE-1 cells were either transfected with short hairpin RNA (shRNA) lentivirus (control) or were stably transfected with sh-MACC1 or sh-negative control (sh-NC) lentivirus (Shanghai Shengbo Biomedical Technology Co. Ltd.) using Lipofectamine 2000 (Shanghai Shengbo Biomedical Technology Co. Ltd.). The shRNA sequences used were as follows: MACC1, forward: 5’- GAGTGCTCACTATGGAAATAA -3’; reverse: 5’- TTATTTCCATAGTGAGCACTC -3’;

NC, forward: 5’-GTTCTCCGAACGTGTCACGTA-3; reverse: 5’-CAAGAGGCTTGCACAGTGCAT-3’’.

### Western Blot Analysis

The total protein was extracted from HNE-1 cell lysates using RIPA buffer (Shanghai Beyotime Biotechnology) and quantified using the BCA kit (Thermo Scientific, USA), and separated by SDS-PAGE before transfer onto a polyvinylidene difluoride membrane. The membrane was then incubated with MACC1 (1:1000; cat# 86290), E-cadherin (1:1000; cat# 20874-1-AP) or vimentin (1:1000; cat# 10366-1-AP) primary antibodies (Shanghai Changjia Biological Co. Ltd.) at 4°C overnight. Next, the blots were incubated with rabbit horseradish peroxidase-conjugated secondary antibody (cat# Sc-2004; 1:3000 dilution; Maixin Biotechnology Company) at room temperature for 1 h. The blots were then visualized using the enhanced chemiluminescence reagent (Shanghai Fusheng Biotechnology Co. Ltd.), and the images were analyzed using the ImageJ software (NIH, USA). Band intensities were normalized against β-actin.

### RNA Extraction and RT-qPCR

TRIzol (Invitrogen, USA) was used for total RNA extraction from 40 of the 128 NPC cases followed by cDNA synthesis (Promega) according to the manufacturer’s instructions. The RT-PCR conditions used were as follows: 95°C for 15 sec; 45 cycles of 95°C for 5 sec with cooling to 60°C for 30 sec; and the absorbance was measured after every elongation cycle. Beta-actin was used as the internal normalization control. The sequences for the primers used were as follows: MACC1, forward: 5’-TTCGGTCAGGAAGAATTG-3 ‘ and reverse: 5’-ATTGTGAAGCAAGTCTGG-3’; vimentin, forward: 5’-CTGAGGGAAACTAATCTG-3’ and reverse: 5’-TTGATAACCTGTCCATCT-3’; E­cadherin, forward: 5’-ATTGCCACATACACTCTC-3’ and reverse: 5’-TGTCATTCTGATCGGTTAC-3’; beta-actin, forward: 5’-CATGTACGTTGCTATCCAGGC-3’ and reverse: 5’- CTCCTTAATGTCACGCACGAT-3’. Relative gene expression levels were calculated using the 2^Δ;ΔCq^ method (35).

### Statistical Analysis

Statistical analyses were performed using the SPSS version 20.0 software (SPSS). Comparisons were performed using the Chi-square test. Univariate survival analyses for the 5-year for overall, relapse-free, metastasis-free and disease-free survival rates were performed using the Kaplan-Meier (KM) survival curves and single-factor log-rank tests. Relapse-free survival is defined as the length of time without any signs or symptoms after treatment completion to the date of recurrence or death from any cause; metastasis-free survival refers to the length of time from the commencement of the cancer treatment wherein the cancer has not spread to other parts of the body; and disease-free survival refers to the period of time during which there are no signs or symptoms of the cancer after successful treatment. The pooled hazard ratios (HRs) with 95% confidence intervals (CIs) were calculated using the multivariate Cox regression analysis model. Correlation analysis was performed using the Spearman’s rank correlation test. Either the date of NPC diagnosis or the commencement of NPC treatment was used to calculate the 5-year survival rates. Values of p<0.05 were deemed statistically significant.

## Results

### MACC1, Vimentin and E-cadherin Protein Levels Are Correlated With TNM and Cancer Staging

We examined the correlation between MACC1 and EMT-related proteins, vimentin and E-cadherin, with clinicopathological factors by immunohistochemistry of the tissue sections of 128 NPC patients. Of the 128 NPC patients, 76 were male and 52 were female, and their average age was 51.79 ± 8.04 years. The median follow-up duration was 60 ± 10 months with a loss rate of 4.7% (6 cases were lost), and the longest follow-up duration was 6 years. Of the 128 patients, 115 patients had UCNT NPC (**Table 1**). There were 38 cases with T1 to T2, 90 cases with T3 to T4, 41 cases with N0 to 1, 87 cases with N2 to 3, 30 cases with stages I to II, and 98 cases with stages III to IVa. MACC1, vimentin, and E-cadherin protein levels in NPC tissues were significantly correlated with T stage (p=0.011), N stage (which is synonymous with lymph node metastasis; p=0.013, 0.011, 0.001, respectively), and cancer stages I to IV (p=0.023, 0.037, 0.022, respectively).

**Table 1:** Relationship between MACC1, E-cadherin, and vimentin expression and the clinicopathological features of NPC patients.

Clinical parameter	n	MACC1				Vimentin				E-cadherin			
	128	positive	negative	χ^2^	p value	positive	negative	χ^2^	p value	positive	negative	χ^2^	p value
Gender													
Male	76	47	29			50	26			37	39		
Female	52	35	17	0.401	0.527	38	14	0.763	0.382	25	27	0.005	0.946
Age (years)													
<50	50	34	16			33	17			23	27		
≥50	78	48	30	0.553	0.457	55	23	0.289	0.591	39	39	0.195	0.659
Histologic type													
UCNT	115	74	41			81	34			54	61		
DNKC	7	4	3			5	2			4	3		
KSCC	6	4	2	0.167	0.92	2	4	3.678	0.159	4	2	1.112	0.574
T stage													
T1-2	38	18	20			20	18			25	13		
T3-4	90	64	26	6.542	0.011	68	22	6.535	0.011	37	53	6.515	0.011
N stage													
N0-1	41	20	21			22	19			29	12		
N2-3	87	62	25	6.119	0.013	66	21	6.395	0.011	33	54	12.004	0.001
TNM stage													
I-II	30	14	16			16	14			20	10		
III-IVa	98	68	30	5.150	0.023	72	26	4.335	0.037	42	56	5.213	0.022

UCNT, undifferentiated carcinoma of nasopharyngeal; DNKC, differentiated non-keratinizing carcinoma; KSCC, keratinizing squamous cell carcinoma.

### MACC1 Protein Level Is Correlated With Vimentin and E-cadherin Protein and mRNA Levels in NPC Tissues

**Figure 1:**
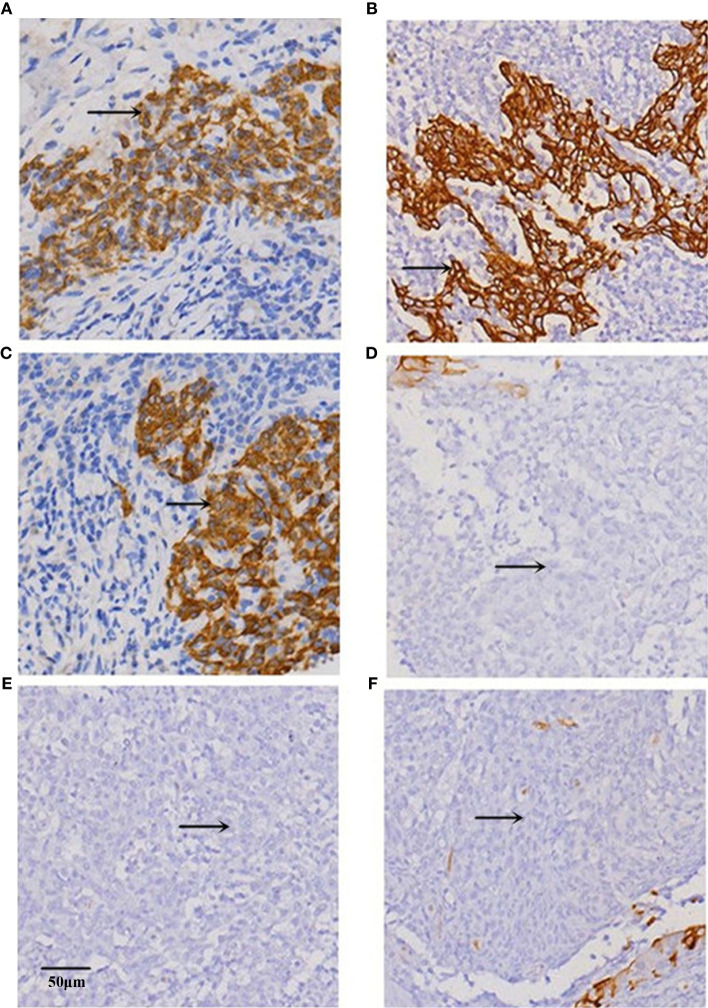
Representative images of immunohistochemical staining for **(A, B)** MACC1, **(C, D)** E-cadherin, and **(E, F)** vimentin proteins in the **(A, C, E)** high and **(B, D, F)** low expression groups of NPC tissues at x200 magnification.

**Figure 2:**
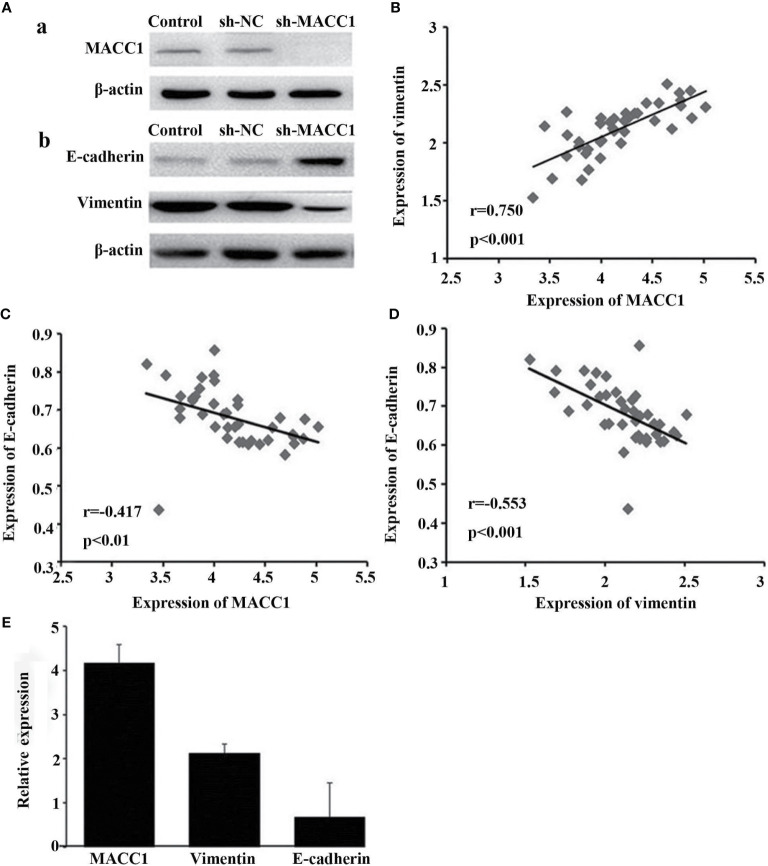
Knockdown of MACC1 inhibits EMT in HNE-1 cells. HNE-1 cells were transfected with sh-MACC1 or sh-NC or were not transfected with shRNA (control). **(A, a)** Western blot showing MACC1 shRNA knockdown in HNE-1 cells. **(A, b)** Western blot showing E-cadherin and vimentin expression in sh-MACC1-transfected HNE-1 cells. **(B)** Correlation analysis of MACC1 and vimentin levels (r=0.750, p<0.001). **(C)** Correlation analysis of MACC1 and E-cadherin levels (r=-0.417, p<0.01). **(D)** Correlation analysis of vimentin and E-cadherin levels (r=-0.553, p<0.001). **(E)** Relative expression of MACC1,vimentin and E-cadherin mRNA.

Given the role of vimentin and E-cadherin proteins in mediating EMT, with the onset of EMT characterized by upregulation of the former and downregulation of the latter (19), we examined their correlation with MACC1 level in NPC. Immunohistochemistry of tissues from 128 NPC patients revealed localization of MACC1 and vimentin to the cytoplasm and cell membrane, and E-cadherin to the cell membrane only (**Figure 1**). Brown granules of MACC1, E-cadherin and vimentin were observed for the high expression groups while light yellow granules were noted for the low expression groups (**Figure 1**). The percentages of cells expressing MACC1, vimentin, and E-cadherin proteins in the 128 NPC samples were 64.06, 68.75, and 48.44%, respectively. Spearman’s rank correlation analysis revealed the positive correlation between MACC1 and vimentin levels (r=0.713, P<0.001), but negative correlation between MACC1 and E-cadherin levels (r=-0.694, P<0.001) as well as vimentin and E-cadherin level (r=-0.615, P<0.001) (data not shown). Immunoblotting following shRNA knockdown of MACC1 in the NPC cell line HNE-1 led to increased E-cadherin but decreased vimentin levels (**Figure 2**), indicating the association between MACC1 and EMT in NPC. Consistent with the results from western blot analysis, RT-qPCR demonstrated the positive correlation between MACC1 and vimentin mRNA levels (MACC1: 4.170 ± 0.423, vimentin: 2.120 ± 0.218; r=0.750, P<0.001) but negative correlation with the E-cadherin mRNA level (**Figure 2**).

### MACC1 and EMT-Related Proteins Are Correlated With NPC Patient Survival

**Figure 3:**
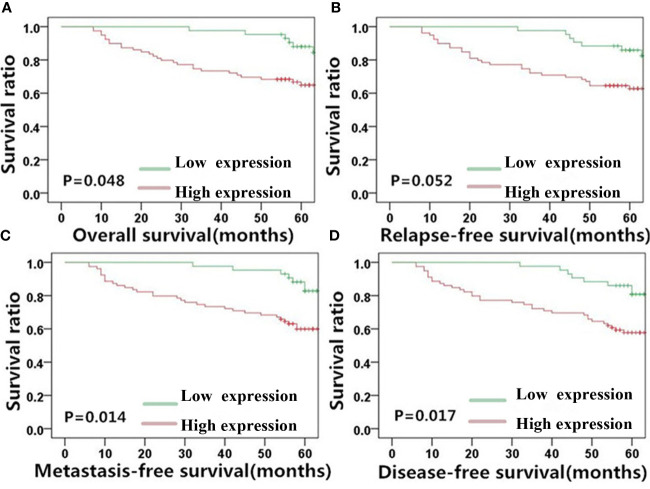
MACC1 level is correlated with NPC patient survival outcome. Kaplan-Meier survival plots for **(A)** overall survival, **(B)** relapse-free survival, **(C)** metastasis-free survival, and **(D)** disease-free survival of the high (red lines) and low (green lines) MACC1 expression groups. MACC1, metastasis-associated in colon cancer 1; NPC, nasopharyngeal carcinoma.

**Figure 4:**
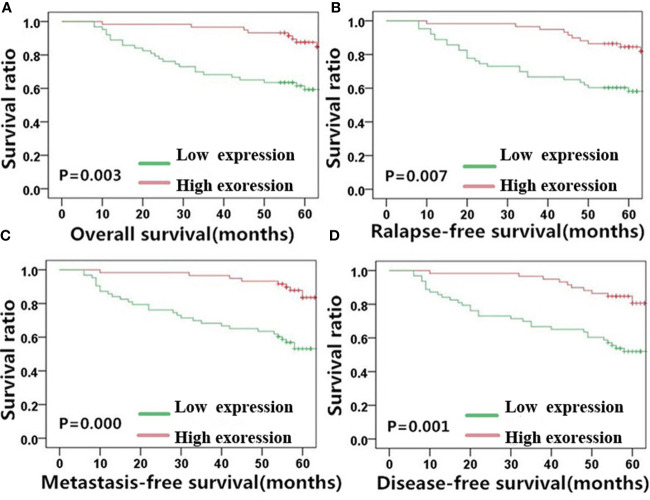
E-cadherin expression is significantly correlated with NPC patient survival outcome. Kaplan-Meier survival plots for **(A)** overall survival, **(B)** relapse-free survival, **(C)** metastasis-free survival, and **(D)** disease-free survival of the high (red lines) and low (green lines) E-cadherin expression groups. NPC, nasopharyngeal carcinoma.

**Figure 5:**
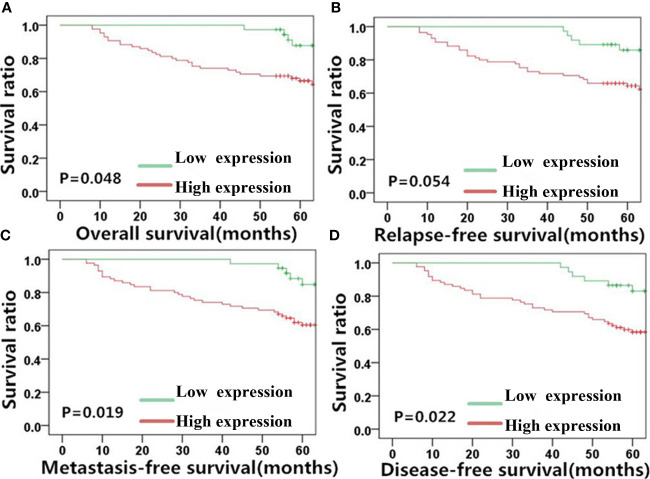
Vimentin expression is significantly correlated with NPC patient survival outcome. Kaplan-Meier survival plots for **(A)** overall survival, **(B)** relapse-free survival, **(C)** metastasis-free survival, and **(D)** disease-free survival of the high (red lines) and low (green lines) vimentin expression groups. NPC, nasopharyngeal carcinoma.

Given the observed correlation between the levels of MACC1 and the EMT-related proteins, E-cadherin and vimentin (**Figure 1**), we determined their association with NPC patient survival (5-year overall, relapse-free, metastasis-free, and disease-free survival rates) using KM survival curves (**Figures 3**–**5**). The 128 NPC patient cohort was grouped according to protein type (MACC1, E-cadherin and vimentin) and protein expression (high and low expression). At the time of the last follow-up in June 2017, 12 patients had local recurrence (9.3%), of which 7 patients had NPC relapse and 5 patients had tumor recurrence at cervical lymph nodes, 25 patients had distant metastasis (19.5%), and 36 patients had died (28%). Among the deaths, 30 cases died from recurrent NPC, and 6 cases died from other tumors. KM survival analysis confirmed the correlation between MACC1, E-cadherin, and vimentin, and NPC patient survival outcome (**Figures 3**–**5**). The percentage of patients who survived throughout the study duration of 5 years decreased with time for the high MACC1 group, high vimentin group, and low E-cadherin group. Analysis of these 3 groups using the Pearson’s Chi-square test revealed that high MACC1 level was significantly correlated with a poor 5-year overall survival rate (63.7%, χ^2^ = 3.915, p=0.048), consistent with the results from a previous report (23). MACC1 overexpression was also closely associated with the 5-year metastasis-free survival rate (60.0%, χ^2^ = 5.991, p=0.014) and 5-year disease-free survival rate (57.0%, χ^2^ = 5.73, p=0.017) but not with the 5-year relapse-free survival rate (60.8%, χ^2^ = 3.772, p=0.052). Patients in the MACC1-negative group showed better 5-year overall, relapse-free, metastasis-free, and disease-free survival rates of 70.4, 68.8, 70.4, and 68.8% respectively.

Similar results as MACC1 was noted for vimentin. High vimentin expression was significantly correlated with a poor 5-year overall survival rate (62.6%, χ^2^ = 3.898, p=0.048), poor 5-year metastasis-free survival rate (59.5%, χ^2^ = 5.494, p=0.019), and poor 5-year disease-free survival rate (56.6%, χ^2^ = 5.211, p=0.022), but not with the 5-year relapse-free survival rate (60.7%, χ^2^ = 3.724, p=0.054). Patients belonging to the vimentin-negative group showed better 5-year overall, relapse-free, metastasis-free, and disease-free survival rates of 72.3, 69.6, 71.3, and 69.7% respectively.

E-cadherin, on the other hand, showed an inverse correlation with NPC survival compared with MACC1 and vimentin. Negative E-cadherin expression was significantly correlated with a poor 5-year overall survival rate (57.3%, χ^2^ = 8.567, p=0.003), poor 5-year relapse-free survival rate (56.4%, χ^2^ = 7.220, p=0.007), poor 5-year metastasis-free survival rate (53.0%, χ^2^ = 12.711, p<0.001), and poor 5-year disease-free survival rate (52.0%, χ^2^ = 10.889, p=0.001). Patients in the E-cadherin-negative group showed better 5-year overall, relapse-free, metastasis-free, and disease-free survival rates of 73.9, 70.6, 73.9, and 70.6%, respectively.

### Prognostic Risk Factors of NPC Survival

Cox regression analyses were performed to assess the influence of MACC1, vimentin, and E-cadherin levels and other clinicopathological factors on patient survival outcomes. Univariate analysis revealed that T, N and cancer staging from stage I to IV were significantly correlated with poor overall, relapse-free, metastasis-free, and disease-free survival (HR>2, p<0.05; **Tables 2** **–** **5**). High expression of MACC1 and vimentin and negative expression of E-cadherin were also shown to be prognostic risk factors for NPC survival outcome (P<0.05; **Table 2**). Multivariate analysis showed that T staging (P=0.049) and E-cadherin (P=0.022) were independent risk factors affecting overall survival (**Table 2**). N staging (P=0.011) was an independent risk factor for relapse-free survival (**Table 3**). TNM staging and E-cadherin were independent risk factors (P<0.05) affecting non-metastatic survival and disease-free survival (**Tables 4** and **5**).

**Table 2:** Univariate and multivariate analyses of prognostic factors for 5-year overall survival of NPC patients.

Parameters	Univariate analysis			Multivariate analysis		
	HR	95% CI	p value	HR	95% CI	p value
Gender						
Male vs. female	0.842	0.426-1.663	0.620			
Age						
<50 vs. ≥50	0.901	0.456-1.779	0.763			
Histologic type						
UCNT vs. DNKC vs. KSCC	0.448	0.155-1.534	0.220			
T stage						
T1-2 vs. T3-4	3.027	1.237-7.406	0.011	2.479	1.002-6.131	0.049
N stage						
N0-1 vs. N2-3	2.672	1.158-6.165	0.021			
TNM stage						
I–II vs. III–IVa	3.457	1.204-9.924	0.021			
MACC1						
positive vs. negative	0.467	0.219-0.995	0.049			
Vimentin						
positive vs. negative	0.432	0.169-0.988	0.047			
E-cadherin						
positive vs. negative	2.729	1.340-5.556	0.006	2.326	1.1.30-4.789	0.022

HR, hazard ratio; CI, confidence interval; UCNT, undifferentiated carcinoma of nasopharyngeal; DNKC, differentiated non-keratinizing carcinoma; KSCC, keratinizing squamous cell carcinoma.

**Table 3:** Univariate and multivariate analyses of prognostic factors for 5-year relapse-free survival of NPC patients.

Parameter	Univariate analysis			Multivariate analysis		
	HR	95% CI	p value	HR	95% CI	p value
Gender						
Male vs. female	0.915	0.480-1.745	0.787			
Age						
<50 vs. ≥50 years	1.126	0.585-2.166	0.723			
Histologic type						
UCNT vs. DNKC vs. KSCC	0.459	0.146-1.444	0.183			
T stage						
T1-2 vs. T3-4	2.701	1.173-6.221	0.02			
N stage						
N0-1 vs. N2-3	2.922	1.278-6.681	0.011	2.922	1.278-6.681	0.011
TNM stage						
I-II vs. III-IVa	3.737	1.310-10.658	0.014			
MACC1						
Positive vs. negative	0.488	0.238-1.004	0.051			
Vimentin						
Positive vs. negative	0.459	0.211-0.999	0.05			
E-cadherin						
Positive vs. negative	2.393	1.228-4.664	0.01			

HR, hazard ratio; CI, confidence interval; UCNT, undifferentiated carcinoma of nasopharyngeal; DNKC, differentiated non-keratinizing carcinoma; KSCC, keratinizing squamous cell carcinoma.

**Table 4:** Univariate and multivariate analyses of prognostic factors for 5-year metastasis-free survival of NPC patients.

Parameter	Univariate analysis			Multivariate analysis		
	HR	95% CI	p value	HR	95% CI	p value
Gender						
Male vs. female	0.898	0.473-1.704	0.743			
Age						
<50 vs. ≥50 years	1.038	0.547-1.970	0.908			
Histologic type						
UCNT vs. DNKC vs. KSCC	0.459	0.145-1.450	0.185			
T stage						
T1-2 vs. T3-4	2.720	1.187-6.234	0.018			
N stage						
N0-1 vs. N2-3	2.898	1.274-6.596	0.011			
TNM stage						
I-II vs. III-IVa	3.719	1.308-10.570	0.014	2.942	1.027-8.432	0.045
MACC1						
Positive vs. negative	0.402	0.191-0.846	0.016			
Vimentin						
Positive vs. negative	0.380	0.168-0.859	0.020			
E-cadherin						
Positive vs. negative	3.241	1.617-6.499	0.001	2.803	1.389-5.658	0.004

HR, hazard ratio; CI, confidence interval; UCNT, undifferentiated carcinoma of nasopharyngeal; DNKC, differentiated non-keratinizing carcinoma; KSCC, keratinizing squamous cell carcinoma.

**Table 5:** Univariate and multivariate analyses of prognostic factors for 5-year disease-free survival.

Parameter	Univariate analysis			Multivariate analysis		
	HR	95% CI	p value	HR	95% CI	p value
Gender						
Male vs. female	0.964	0.523-1.778	0.907			
Age						
<50 vs. ≥50 years	1.059	0.570-1.965	0.857			
Histologic type						
UCNT vs. DNKC vs. KSCC	0.431	0.136-1.366	0.153			
T stage						
T1-2 vs. T3-4	2.473	1.134-5.397	0.023			
N stage						
N0-1 vs. N2-3	3.165	1.400-7.153	0.006			
TNM stage						
I-II vs. III-IVa	4.011	1.419-11.343	0.009	3.271	1.147-9.329	0.027
MACC1						
Positive vs. negative	0.425	0.209-0.863	0.018			
Vimentin						
Positive vs. negative	0.407	0.189-0.878	0.022			
E-cadherin						
Positive vs. negative	2.812	1.465-5.398	0.002	2.403	1.244-4.642	0.009

HR, hazard ratio; CI, confidence interval; UCNT, undifferentiated carcinoma of nasopharyngeal; DNKC, differentiated non-keratinizing carcinoma; KSCC, keratinizing squamous cell carcinoma.

## Discussion

MACC1 has been implicated in cancer malignancy and poor disease prognosis in several cancer types (7, 16, 25, 26), and its downregulation was found to reduce tumorigenicity (12). Decrease in MACC1 level can inhibit NPC cell proliferation and stemness *via* the Akt/β-catenin pathway (11) and Smad2/MACC-1-AS1 (antisense long non-coding RNA (lncRNA) MACC-1-AS) axis (36), respectively. Consistent with previous findings from Liang et al. (23), we confirmed that MACC1 was correlated with TNM classification, cancer staging, and poor NPC survival outcomes by immunohistochemistry and survival analyses in the present study. However, the Liang et al. study reported a much lower 5-year overall survival rate of 45.9% for patients with high MACC1 NPC (23) compared to 63.7% calculated in this study. The intrinsic characteristics of each cohort group, which may differ in treatment methods and cancer severity, may account for this difference. Consistent with the significant association between MACC1 expression and the metastasis-free survival rate (7), we confirmed this correlation in NPC in this study.

EMT has been implicated as the underlying mechanism of tumorigenicity and progression of cancers including colorectal and cervical cancer (20, 29, 30, 32, 33). As EMT is characterized by the upregulation of vimentin and the loss of E-cadherin (25, 31, 32), we selected these two cell adhesion markers to characterize the relationship between MACC1 and EMT and also their association with NPC malignancy. Although MACC1 is associated with EMT in other cancers (10, 20, 29, 30, 37), their relationship in NPC has not been characterized. In this study, we demonstrated the association of MACC1 and EMT with NPC progression.

Consistent with a previous study on uterine cervical cancer (20), the correlation between MACC1 and the EMT-related proteins, vimentin and E-cadherin, observed in this study indicate the association between MACC1 and EMT in NPC, thus adding NPC to the list of cancers previously reported to involve EMT (31, 33). In addition, MACC1, E-cadherin and vimentin levels were correlated with cancer staging and TNM tumor classification. Taken together, our results propose these three proteins as potential NPC prognostic biomarkers. KM survival analyses suggested MACC1 and vimentin as markers for predicting overall, metastasis-free, and disease-free survival in NPC patients but are less reliable for predicting relapse-free survival, whereas E-cadherin, could serve as a prognostic marker for overall, relapse-free, metastasis-free, and disease-free survival.

Given the known geographical and ethnic bias of NPC (1, 3), the conclusions in the present study may not be representative. Including data of patients from other hospitals in Southern China would augment the reliability of the conclusions and ensure more comprehensive data coverage. In addition, including data of patients of other races such as from Southeast Asian, Mediterranean and Arctic populations would add confidence and accuracy to the conclusions of MACC1’s potential as a NPC prognostic biomarker. Other potential limitations of this study include differences in the therapy administered to each of the patients and the heterogeneity introduced by the cut-off values applied to stratify the patients into high and low expression groups for MACC1 and the two EMT-related proteins.

The standard treatment method for NPC is radiotherapy and this can be combined with chemotherapy especially for patients with advanced tumor growth (38). The nasopharynx is located in close proximity to vital organs in the head and neck area, thus imposing limitations to treatment methods such as surgery. This also poses limitations to the radiation dosage for radiotherapy, thus making NPC treatment challenging. Therefore, there is an imperative need for biomarker discovery to enable early and accurate NPC diagnosis and prognosis. Active areas of research are directed at improving locoregional control of the tumor, improving the intrinsic radiosensitivity of NPC tumor cells, reducing late-stage toxicity that may arise from tumor secretions, and seeking effective radiosensitive targets. Tumor cells with EMT-like properties had demonstrated tolerance to radiotherapy and chemotherapy treatments (33, 39). Of note, loss of E-cadherin and cell type transformation led to tumor radioresistance (40). Given our observation of the significant association between MACC1, EMT and NPC survival outcome, subsequent research should be directed toward characterizing the relationship between MACC1, EMT and NPC radioresistance.

In conclusion, the present study confirms the association between MACC1 expression and NPC malignancy (23), and suggests the potential of MACC1 as a prognostic biomarker as well as a molecular target protein in NPC treatment. Furthermore, our study revealed significant correlations between the levels of MACC1 and two EMT markers in NPC, suggesting that MACC1 may mediate EMT thereby promoting NPC malignancy.

## Data Availability Statement

The original contributions presented in the study are included in the article/supplementary material. Further inquiries can be directed to the corresponding author.

## Ethics Statement

The studies involving human participants were reviewed and approved by Ethics Committee of the First People’s Hospital of Chenzhou. The patients/participants provided their written informed consent to participate in this study.

## Author Contributions

HC performed the experiments, collected and analyzed the data, and wrote the manuscript. LZ, YL, and JX analyzed the immunohistochemistry data. LZ, YL, and JX collected the patient data. LC designed and supervised the study. All authors contributed to the article and approved the submitted version.

## Funding 

This work was supported by the in-house project funding support of the First People’s Hospital of Chenzhou (N2018-003).

## Conflict of Interest

The authors declare that the research was conducted in the absence of any commercial or financial relationships that could be construed as a potential conflict of interest.
